# Multidrug-resistant non-typhoidal Salmonella and Escherichia coli in imported poultry products in the Maldives

**DOI:** 10.1099/acmi.0.001114.v3

**Published:** 2026-03-11

**Authors:** Zeeniya Kamil, Wing-Sze Lau, Shazla Mohamed, Richard A. Stabler

**Affiliations:** 1 Department of Environment and Natural Science, Faculty of Engineering, Science and Technology, The Maldives National University, Malé, Maldives; 2 Department of Infection Biology, London School of Hygiene and Tropical Medicine, London, UK; 3 Research Development Office, The Maldives National University, Malé, Maldives

**Keywords:** antimicrobial resistance (AMR), *Escherichia coli*, Maldives, multidrug resistance (MDR), non-typhoidal *Salmonella* (NTS), poultry

## Abstract

Antimicrobial resistance (AMR) increasingly compromises food safety and public health worldwide. Poultry products are major vectors for AMR bacteria in the food supply. We conducted the first preliminary survey of non-typhoidal *Salmonella* (NTS) and *Escherichia coli* on imported poultry in the Maldives. A total of 30 frozen whole chicken samples (15 processed as whole and 15 separated into meat and skin) and 3 pooled egg samples (10 eggs per pool) were obtained from supermarkets and grocery stores in Greater Malé between June 2022 and July 2022. Standard culture methods (Food and Drug Administration Bacteriological Analytical Manual) were used to isolate NTS and *E. coli*, and isolates were tested for susceptibility to five antibiotics (ampicillin, ceftriaxone, ciprofloxacin, tetracycline and trimethoprim-sulphamethoxazole) by disc diffusion [European Committee on Antimicrobial Susceptibility Testing (EUCAST) guidelines]. NTS was recovered from 10 of 30 (33.3%) chicken samples, predominantly from skin (9/15) versus meat (3/15); *E. coli* was found in 15 of 30 (50%) samples, more often in meat. One pooled egg sample (33%) was positive for *E. coli*. Among 13 NTS isolates, 69% (9/13) were resistant to tetracycline, and 38% (5/13) to ciprofloxacin, ampicillin and ceftriaxone. Thirty-eight per cent (5/13) of NTS were classified by EUCAST as susceptible, increased exposure to ciprofloxacin. Overall, 9 of 13 (69.2%) NTS isolates were multidrug-resistant (MDR; non-susceptible to ≥3 classes). In *E. coli,* resistance was most common to ampicillin (8/19; 42.1%), followed by tetracycline (5/19; 26.3%), trimethoprim-sulphamethoxazole (4/19; 21.1%), ciprofloxacin (1/19; 5.3%) and ceftriaxone (1/19; 5.3%), with 26.3% (5/19) of *E. coli* being MDR. These results indicate a substantial prevalence of MDR foodborne bacteria in imported poultry and underscore critical food safety and One Health concerns. Strengthened microbiological surveillance, risk-based import inspection and enhanced regulatory coordination (aligned with the Maldives’ AMR Action Plan) are urgently needed to protect public health.

## Data Summary

All raw data are included in supplementary files.

## Introduction

Antimicrobial resistance (AMR) is a rapidly growing global health concern, projected to cause up to ten million deaths annually by 2050 if left unaddressed [1]. It compromises the efficacy of essential medicines, increasing the risk of prolonged illness, treatment failure and mortality. While AMR has often been associated with clinical misuse, the overuse of antibiotics in food-producing animals, particularly in intensive poultry farming, has emerged as a major contributor [2]. In many countries, antimicrobials are routinely administered for growth promotion, disease prevention and therapeutic purposes [3]. These practices create selective pressure that enables resistant bacteria to proliferate within the animal microbiota and spread across populations [4]. Humans can acquire these resistant bacteria via contaminated food, direct contact with animals or environmental pathways, making AMR an essential One Health challenge [5].

Poultry, one of the most widely consumed animal proteins globally and in the Maldives, plays a central role in this transmission. Non-typhoidal *Salmonella* (NTS) and *Escherichia coli* are among the most isolated foodborne pathogens from poultry. NTS causes an estimated 150 million infections and 60,000 deaths globally each year and remains a leading cause of foodborne illness and hospitalization [6,7]. Invasive strains resistant to fluoroquinolones or third-generation cephalosporins are particularly concerning due to their association with severe disease and treatment failure [8]. Although *E. coli* is typically a harmless commensal, its presence in poultry products indicates faecal contamination and poor hygiene practices. Moreover, antimicrobial-resistant *E. coli* strains are increasingly recognized as reservoirs of transferable resistance genes [5,9].

In recognition of these risks, the World Health Organisation (WHO) promotes a One Health approach to AMR**,** advocating for integrated surveillance across human, animal and environmental health sectors [10]. However, many low- and middle-income countries, including Small Island Developing States (SIDS) such as the Maldives, face infrastructural, regulatory and technical barriers to implementing comprehensive AMR surveillance, especially in food systems.

The Maldives, a SIDS with limited domestic poultry production, relies heavily on imported poultry products, primarily from Brazil, India, Sri Lanka and Turkey, which have reported widespread microbial contamination and multidrug-resistant (MDR) pathogens in poultry meat and eggs [11,13]. This dependence presents critical public health challenges, particularly given national constraints in laboratory capacity, trained personnel, food safety regulation and intersectoral AMR coordination. The Maldives’ National Action Plan on Antimicrobial Resistance (2024–2029) identifies these gaps as key barriers to effective AMR response [14]. Additionally, the country’s tropical climate and documented hygiene lapses in food handling and storage in urban centres such as Malé further elevate the risk of foodborne AMR transmission [15]. There is a significant burden on the Maldives’ healthcare system and economy from diarrhoeal diseases caused by zoonotic and foodborne pathogens such as NTS and pathogenic *E. coli*; a World Bank report indicated that between 2009 and 2017, the proportion of children under 5 receiving rehydration therapy for diarrhoea in the Maldives increased from 63% to 75%.

Despite the growing urgency to address AMR, there is a lack of baseline data on the prevalence and resistance profiles of foodborne pathogens in imported poultry in the Greater Malé. This study addresses this gap by investigating the presence and AMR characteristics of NTS and *E. coli* in frozen whole chicken and composite egg samples sold in Greater Malé. As the first study of its kind in the country, the findings provide essential baseline data to support future AMR surveillance efforts, inform food safety risk assessments and guide the development of context-specific mitigation strategies under the One Health framework.

## Methods

### Study area and sampling strategy

This study was conducted in the Greater Malé region, comprising Malé, Hulhumalé and Villingili within the Republic of the Maldives between 7 June 2022 and 18 July 2022 (42 days). A total of 30 frozen whole chicken samples and three composite fresh egg samples (30 eggs per pool) were collected by a single researcher from 16 supermarkets and 14 grocery stores. Retail outlets were purposively selected to capture diversity in retail types, including corner shops, small independent grocery shops and large chain supermarkets. Where chain retailers had multiple branches, different branches were included. The selected outlets represent variations in sourcing and storage practices. Supermarkets were defined as large retail chains with centralized supply chains, whereas grocery stores were small, independently operated outlets.

### Microbiological analysis

#### Sample processing and enrichment

The chicken samples were split into two groups: 15/30 were processed as a whole carcass intact, and the remaining 15 were aseptically separated into meat and skin portions to compare microbial contamination levels by tissue type. All samples were processed on the day of purchase to minimize cross-contamination.

Microbiological analysis was performed in accordance with the U.S. Food and Drug Administration’s Bacteriological Analytical Manual [16]. Frozen chicken samples were stored at room temperature overnight until thawed. The external surface of whole chicken sample packaging was wiped thoroughly with 70% ethanol, and the packaging was cut open aseptically with sterilized scissors in a sterile environment. Chicken meat and skin samples were cut out aseptically from the breast, thigh and drumstick using sterile knives and forceps. Batches of ten eggs were pooled together as one composite sample. In an aseptic environment, egg shells were cracked open with a sterile knife, and the contents were emptied into a sterile stomacher bag. For both chicken and egg samples, 25 g portions were homogenized in 225 ml of buffered peptone water (BPW) using a Stomacher^®^ 400 Circulator (Seward Ltd., UK) at 230 r.p.m. for 60–120 s. Homogenates were incubated at 37 °C for 18–24 h.

For *Salmonella* spp., 0.1 ml of pre-enriched BPW culture was transferred into 10 ml of Rappaport–Vassiliadis enrichment broth and incubated at 42 °C for 18–24 h. A 10 µl loopful was then streaked onto Xylose Lysine Deoxycholate (XLD) agar plates and incubated under aerobic conditions.

For *E. coli*, a 10 µl loopful of the BPW pre-enrichment was directly streaked onto MacConkey agar. Presumptive colonies from both media were further purified on fresh agar plates and subjected to confirmatory tests.

### Identification of bacterial isolates

Presumptive *Salmonella* and *E. coli* isolates were confirmed using standard biochemical methods, including Triple Sugar Iron slants, indole production in tryptone water, citrate utilization, urease activity, catalase and oxidase testing and lactose fermentation on MacConkey agar. *Salmonella* spp. isolates were further confirmed by slide agglutination using polyvalent O and H antisera (Pro-laboratory Diagnostics, UK). One confirmed isolate per culture-positive sample was retained for antimicrobial testing.

Microscopy was used to verify Gram-negative rod morphology. All procedures followed Food and Drug Administration's Bacteriological Analytical Manual (FDA BAM) protocols for foodborne pathogen identification. Reference strains *E. coli* ATCC 25922 and *Salmonella enterica* serovar Typhimurium ATCC 51812 were used as quality control organisms.

### Antimicrobial susceptibility testing

Antimicrobial susceptibility testing was performed using the Kirby–Bauer disc diffusion method following the European Committee on Antimicrobial Susceptibility Testing (EUCAST) guidelines (v12.0, 2023) (Supplementary data 1, available in the online Supplementary Material). The antibiotic panel included ampicillin (10 µg), ceftriaxone (30 µg), ciprofloxacin (5 µg), tetracycline (30 µg) and trimethoprim-sulphamethoxazole (SXT, 25 µg). Zone diameters were measured in millimetres and interpreted as susceptible (S) or resistant (R) per standard guidelines. For *Salmonella* and ciprofloxacin, Clinical and Laboratory Standards Institute (CLSI) interpretive criteria were used to define resistant, susceptible, increased exposure and susceptible (Supplementary data 1). MDR was defined as non-susceptible to ≥3 antimicrobial classes.

### Statistical analysis

Descriptive statistics were computed using STATA v17.0 (StataCorp, USA). Frequencies and proportions were calculated for the prevalence of contamination and AMR. Associations between contamination patterns (e.g. meat versus skin; supermarket versus grocery store) were assessed using Pearson’s chi-square or Fisher’s exact tests, as appropriate. A *P*-value of <0.05 was considered statistically significant. Where applicable, 95% confidence intervals were reported to describe the precision of the estimates.

## Results

### Prevalence of NTS

NTS was detected in 33% (10/30) of the frozen chicken samples collected from retail outlets in Greater Malé (Table 1). Among the subset of skin and meat samples processed separately, NTS was isolated more frequently from skin (9/15; 60%) than from meat (3/15; 20%). In contrast, only 1 of the 15 whole chicken samples (7%) tested positive for NTS. However, the differences in prevalence between skin and meat were not statistically significant (*P*>0.05).

**Table 1.** Prevalence of NTS and *E. coli* in poultry and egg samples. Sample counts refer to the number of pooled or processed units tested. Some samples yielded both organisms

Sample type	No. tested	NTS positive *n* (%)	*E. coli* positive *n* (%)
Whole chicken	15	1 (7%)	6 (40%)
Skin (separated)	15	9 (60%)	5 (33%)
Meat (separated)	15	3 (20%)	7 (47%)
Composite egg pool	3	0 (0%)	1 (33%)

E. coli, Escherichia coli; No., number; NTS, Nontyphoidal Salmonella.

None of the three composite egg samples (each representing a pooled set of ten eggs) tested positive for NTS. When frozen chicken samples were stratified by retail source, NTS prevalence was similar between supermarkets (5/16; 31%) and grocery stores (5/14; 36%), with no significant difference observed (*P*>0.05) (Table 2).

**Table 2.** Prevalence of NTS and *E. coli* in frozen chicken samples by retail source

Type of shop	*n*	NTS *n* (%)	*E. coli* *n* (%)
Supermarkets	16	5 (31%)	6 (38%)
Grocery stores	14	5 (36%)	9 (64%)
**Total**	**30**	**10 (33%)**	**15 (50%)**

E. coli, Escherichia coli; NTS, Non-typhoidal Salmonella.

### Prevalence of *E. coli*

*E. coli* was detected in 50% (15/30) of the frozen chicken samples (Table 1). Among the subset of 15/30 frozen chicken samples processed and analysed separately, *E. coli* was isolated from 7 meat samples (47%) and 5 skin samples (33%). Among the 15 whole chicken samples, 6 (40%) tested positive for *E. coli*. However, as with NTS, no significant differences in *E. coli* prevalence were observed between meat or skin (*P*>0.05).

*E. coli* was detected in one of the three composite egg samples (33%). In frozen chicken samples, *E. coli* contamination was more frequent in grocery store samples (9/14; 64%) than in supermarket samples (6/16; 38%), but this difference was not statistically significant (*P*>0.05) (Table 2).

### AMR profiles

#### Resistance in NTS isolates

**Fig. 1.**
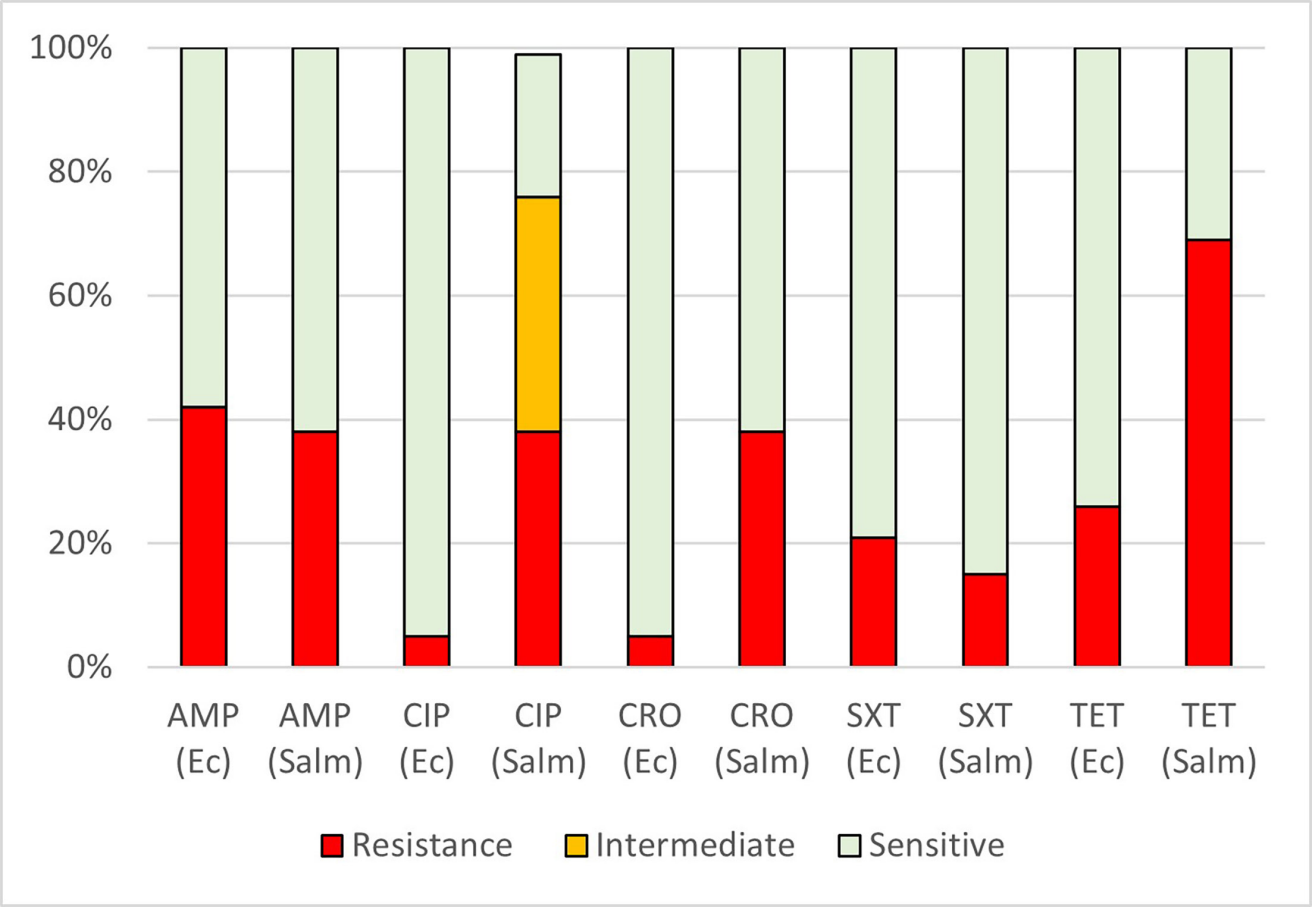
Resistance to antibiotics in poultry isolates. AMP, CIP, CRO, SXT, TET, Ec, Salm, resistant (red), ciprofloxacin ‘susceptible increased exposure’ (EUCAST) (orange), sensitive (green). AMP, ampicillin; CIP, ciprofloxacin; CRO, ceftriaxone; Ec, *E. coli*; Salm, non-typhoidal *Salmonella*; TET, tetracycline.

Thirteen NTS isolates from frozen chicken and egg samples were tested for antimicrobial susceptibility (Fig. 1). High resistance rates were observed for tetracycline (9/13; 69%). Ciprofloxacin resistance was present in 5/13 (38%), and 5/13 (38%) were intermediate (susceptible, increased exposure – EUCAST). Resistance to ampicillin and ceftriaxone was detected in five isolates each (38%), while resistance to SXT was observed in three isolates (23%) (Fig. 1). Based on the definition of MDR as non-susceptibility to three or more antibiotic classes, 69% (9/13) of NTS isolates were classified as MDR. Among these, four isolates (31%) were resistant to three classes, and three isolates (23%) were resistant to four classes.

#### Resistance in *E. coli* isolates

Nineteen *E. coli* isolates from frozen chicken and egg samples were tested for antimicrobial susceptibility. The highest resistance was observed to ampicillin (8/19; 42%), followed by tetracycline (5/19; 26%), SXT (4/19; 21%), ciprofloxacin (1/19; 5%) and ceftriaxone (1/19; 5%) (Fig. 1). Overall, 5 of the 19 isolates (26%) met the criteria for MDR. Two isolates (11%) were resistant to three or more antibiotic classes, and three isolates (16%) were resistant to two.

## Discussion

This study provides the first preliminary investigation of microbial contamination and AMR in imported poultry products sold in the Maldives. The incidence of NTS was 33%, and *E. coli* was 50% of frozen chicken samples, indicating a substantial microbial burden in widely consumed imported food items.

The NTS prevalence observed in this study was consistent with findings from several key poultry-exporting countries. For example, *Salmonella* contamination was reported at 34.3% in raw chicken meat in Brazil [4], 38.2% in poultry products in Türkiye [17] and 78% in chicken meat in Sri Lanka [18]. These findings underscore the widespread and persistent challenge of *Salmonella* contamination in poultry supply chains across various regions.

Similarly, the prevalence of *E. coli* in frozen chicken samples aligns with international trends. In Sri Lanka, *E. coli* was detected in 66.8% of chicken meat and organ samples from retail outlets [19], while in southern Brazil, 58.7% of retail chicken samples tested positive [20]. Other studies report prevalence rates ranging from ∼48% to over 74% in retail poultry products globally, indicating that *E. coli* contamination is a consistent and widespread issue [21,22]. The detection of *E. coli* in one of three pooled egg samples further highlights potential microbial contamination during transport or retail handling and points to the need for improved hygiene practices across the supply chain.

Microbial distribution across sample types also warrants attention. NTS was more frequently found on chicken skin than meat, whereas *E. coli* appeared more often on meat than skin. Although these differences were not statistically significant due to the small sample size, they highlight possible variation in contamination dynamics. Similarly, no statistically significant differences in contamination rates of *E. coli* and NTS from frozen chicken samples were observed between supermarkets and grocery stores; however, a slightly higher prevalence was noted in grocery stores, suggesting that a larger sample size is needed to confirm this trend.

Fluoroquinolones and third-generation cephalosporins are clinically important antimicrobials for human medicine and are often used as first-line treatments for salmonellosis in adults and children, respectively [23,24]. The AMR profiles of NTS observed in this study are particularly concerning. Among NTS isolates, resistance to tetracycline (69%) was notably high, with 69% of isolates classified as MDR.

Comparable resistance trends have been observed in major poultry-exporting countries. In Brazil, a nationwide study found 89.0% ciprofloxacin resistance and 76.1% resistance to third-generation cephalosporins, while a 20-year meta-analysis reported 42.5% resistance to nalidixic acid in poultry-associated NTS isolates [25,26]. The continued use of fluoroquinolones and ceftiofur, a third-generation cephalosporin, in the Brazilian poultry industry contributes significantly to this resistance, exerting selection pressure that favours the persistence and spread of resistant *Salmonella* strains [27].

Globally, studies report high-level AMR in *Salmonella*, for example, 68.7% ciprofloxacin resistance in poultry-associated *Salmonella* in the UAE [28], and found >70% fluoroquinolone resistance in *Salmonella* from Nigerian poultry farms [29]. The World Health Organization classifies ciprofloxacin and third-generation cephalosporins, such as ceftriaxone (38% resistance observed in this study), as critically important antimicrobials [30], and resistance to these agents limits treatment options for invasive infections.

While *E. coli* isolates showed lower resistance to ciprofloxacin (5%) and ceftriaxone (5%), ampicillin (42%) and tetracycline (26%) resistance levels were notable. A total of 26% of *E. coli* isolates were classified as MDR. A 2024 study found that 63.5% of avian pathogenic *E. coli* isolates from Brazilian broilers carried β-lactam resistance genes, while nearly half also harboured resistance to fluoroquinolones and other antibiotic classes, indicating widespread MDR among these strains [20]. Research confirms that *E. coli* isolated from Brazilian poultry exhibit high levels of resistance to both β-lactam antibiotics and fluoroquinolones, raising significant public health concerns [20]. The pattern of resistance is not unique to Brazil, as similar findings have been reported in poultry from other countries, but the high prevalence in Brazil is particularly concerning given its status as a major exporter to the Maldives and globally [11]. The differences in resistance rates observed could be due to the regulations on antimicrobial use in different countries. Another factor could be due to the methodology employed, for instance, molecular identification could identify genes phenotypically silent under laboratory conditions. MDR *E. coli* in food products poses an indirect public health threat due to its potential role as a reservoir of resistance genes that may be horizontally transferred to pathogenic bacteria in the human gut [31,32].

*Salmonella* contamination in poultry varies by region and is strongly influenced by national differences in biosecurity practices, veterinary oversight and antimicrobial stewardship policies. The European Union, through its harmonized surveillance and regulatory frameworks, has achieved a relatively low prevalence of NTS in poultry production [33]. In contrast, countries with less stringent enforcement, such as Brazil, report persistent contamination and the emergence of MDR strains [34]. Genomic surveillance in Brazil has identified high-risk clones of *Salmonella* Heidelberg and *Salmonella* Minnesota carrying resistance to third-generation cephalosporins and fluoroquinolones [11]. These findings highlight the international dissemination of AMR through food trade.

The Maldives’ dependence on poultry imports, limited diagnostic capacity, lack of systematic AMR monitoring in food and tropical climate create a setting vulnerable to the introduction and dissemination of resistant foodborne pathogens. Previous reviews have emphasized the absence of national foodborne disease surveillance systems in the country [35]. By providing the first baseline data on microbial contamination and AMR in imported poultry, this study fills a critical knowledge gap and underscores the need for strengthened regulatory measures, risk-based import inspection and the integration of food safety into the national AMR surveillance agenda under the One Health framework.

This study has several limitations. The sample size was small and cross-sectional, which limits the generalizability of the findings and prevents the assessment of temporal or seasonal variation. Although country-of-origin information was collected for imported poultry products, the small number of samples per country precluded meaningful analysis of contamination or resistance patterns by export source. Additionally, molecular characterization of resistance genes was not performed, which limits insight into specific transmission pathways and the genetic basis of resistance. Only phenotypic resistance profiles were assessed. Nonetheless, these preliminary findings provide a crucial baseline for the Maldives and underscore the need for future, larger scale studies incorporating molecular methods, such as resistance gene typing and whole-genome sequencing, to support risk-based surveillance and inform public health policy.

Despite its limitations, this study provides the first evidence of MDR NTS and *E. coli* in imported poultry products in the Maldives. The findings highlight an urgent need for routine AMR surveillance at ports of entry, improved laboratory capacity for food microbiology and resistance testing, the implementation of risk-based food import policies and the integration of food safety into the national AMR Action Plan under the One Health framework. In SIDS such as the Maldives, where domestic food production is limited and reliance on imports is high, foodborne AMR represents not only a growing public health concern but also a broader threat to national health security. Addressing this issue will require coordinated, cross-sectoral efforts that align regulatory reform, diagnostic investment and international collaboration to strengthen food safety and AMR mitigation in the region.

This preliminary study offers the first evidence of MDR NTS and *E. coli* in imported poultry products sold in the Maldives. While limited in scope, the findings provide important baseline data that underscore the potential public health risks posed by antimicrobial-resistant foodborne pathogens in imported food. As an initial step, the results support the need for targeted enhancements in AMR monitoring and food safety. Specifically, the study highlights the value of piloting routine microbiological screening at key ports of entry, investing in laboratory capacity for foodborne AMR detection and gradually aligning food import oversight with the national AMR Action Plan through a One Health approach. In SIDS like the Maldives – where food imports dominate the national diet – even limited studies such as this play a critical role in informing evidence-based policy. Future research with expanded sampling and molecular analyses is essential to validate these findings and guide broader national interventions.

## Supplementary material

10.1099/acmi.0.001114.v3 Uncited Supplementary Material 1.

10.1099/acmi.0.001114.v3 Uncited Supplementary Material 2.

10.1099/acmi.0.001114.v3 Uncited Supplementary Material 3.

10.1099/acmi.0.001114.v3 Uncited Supplementary Material 4.

## Abbreviations

AMR: antimicrobial resistance
BPW: buffered peptone water
CLSI: Clinical and Laboratory Standards Institute
EUCAST: European Committee on Antimicrobial Susceptibility Testing
MDR: multidrug-resistant
NTS: non-typhoidal *Salmonella*
SIDS: Small Island Developing States
SXT: trimethoprim-sulphamethoxazole
XLD: Xylose Lysine Deoxycholate

## References

[1] O’Neil J (2016). Tackling drug-resistant infections globally: final report and recommendations. Review AMR.

[2] Hoelzer K, Wong N, Thomas J, Talkington K, Jungman E (2017). Antimicrobial drug use in food-producing animals and associated human health risks: what, and how strong, is the evidence?. BMC Vet Res.

[3] Odey TOJ, Tanimowo WO, Afolabi KO, Jahid IK, Reuben RC (2024). Antimicrobial use and resistance in food animal production: food safety and associated concerns in Sub-Saharan Africa. Int Microbiol.

[4] Medeiros MAN, Oliveira DCN de, Rodrigues D dos P, Freitas DRC de (2011). Prevalence and antimicrobial resistance of *Salmonella* in chicken carcasses at retail in 15 Brazilian cities. Rev Panam Salud Publica.

[5] FAO, WHO (2019). Joint FAO/WHO expert meeting in collaboration with OIE on foodborne antimicrobial resistance: role of the environment, crops and biocides.

[6] CDC (2024). Yellow book: Salmonellosis, Nontyphoidal.

[7] Sima CM, Buzilă ER, Trofin F, Păduraru D, Luncă C (2024). Emerging strategies against non-typhoidal *Salmonella*: from pathogenesis to treatment. Curr Issues Mol Biol.

[8] Tack B, Vanaenrode J, Verbakel JY, Toelen J, Jacobs J (2020). Invasive non-typhoidal *Salmonella* infections in sub-Saharan Africa: a systematic review on antimicrobial resistance and treatment. BMC Med.

[9] Tadesse DA, Zhao S, Tong E, Ayers S, Singh A (2012). Antimicrobial drug resistance in *Escherichia coli* from humans and food animals, United States, 1950-2002. *Emerg Infect Dis*.

[10] WHO (2022). Global antimicrobial resistance and use surveillance system (GLASS) report: 2022.

[11] Kipper D, Mascitti AK, De Carli S, Carneiro AM, Streck AF (2022). Emergence, dissemination and antimicrobial resistance of the main poultry-associated *Salmonella* serovars in Brazil. Vet Sci.

[12] Çufaoğlu G, Ambarcioğlu P, Deri̇nöz AN, Ayaz ND (2023). Prevalence, serotype diversity and antibiotic resistance of *Salmonella* in poultry meat and egg in Turkey: a meta-analysis. *J Agr Sci-Tarim Bili*.

[13] Subhasinghe SAIC, Pabasara ABS, Pathiraja PMHM, Karunarathna HMTK, Kalupahana RS (2023). Glimpse into the biosecurity, antimicrobial usage, and antimicrobial resistance of fecal *Escherichia coli* associated with commercial chicken layer farms in a poultry dense area in Sri Lanka. Sri Lanka Vet J.

[14] Ministry of Health Republic of Maldives (2024). National Action Plan and Antimicrobial Resistance 2024-2029.

[15] Halim-Lim SA, Mohamed K, Sukki FM, David W, Ungku Zainal Abidin UF (2023). Food safety knowledge, attitude and practices of food handlers in restaurants in the Maldives. Sustainability.

[16] Andrews WH, Wang H, Jacobson A, Ge B, Zhang G (2024). Bacteriological Analytical Manual.

[17] Dishan A, Hizlisoy H, Onmaz NE, Yildirim Y, Gonulalan Z (2024). Comprehensive analysis of *Salmonella* in poultry meat and products in Türkiye: Prevalence, antibiotic susceptibility and genomic characterisation. Int J of Food Sci Tech.

[18] Weerasooriya G, Dulakshi HMT, de Alwis PS, Bandara S, Premarathne KRPS (2024). Persistence of *Salmonella* and *Campylobacter* on whole chicken carcasses under the different chlorine concentrations used in the chill tank of processing Plants in Sri Lanka. Pathogens.

[19] Ranasinghe RASS, Satharasinghe DA, Anwarama PS, Parakatawella PMSDK, Jayasooriya LJPAP (2022). Prevalence and antimicrobial resistance of *Escherichia coli* in chicken meat and edible poultry organs collected from retail shops and supermarkets of North Western Province in Sri Lanka. J Food Qual.

[20] Pilati GVT, Salles GBC, Savi BP, Dahmer M, Muniz EC (2024). Isolation and characterization of *Escherichia coli* from Brazilian broilers. Microorganisms.

[21] Lee KY, Lavelle K, Huang A, Atwill ER, Pitesky M (2023). Assessment of prevalence and diversity of antimicrobial resistant *Escherichia coli* from retail meats in Southern California. Antibiotics (Basel).

[22] Rahman MM, Husna A, Elshabrawy HA, Alam J, Runa NY (2020). Isolation and molecular characterization of multidrug-resistant *Escherichia coli* from chicken meat. Sci Rep.

[23] Majowicz SE, Musto J, Scallan E, Angulo FJ, Kirk M (2010). The global burden of nontyphoidal *Salmonella gastroenteritis*. Clin Infect Dis.

[24] Hohmann EL (2001). Nontyphoidal salmonellosis. Clin Infect Dis.

[25] Rau RB, Ribeiro AR, dos Santos A, Barth AL (2021). Antimicrobial resistance of *Salmonella* from poultry meat in Brazil: results of a nationwide survey. Epidemiol Infect.

[26] Voss-Rech D, Potter L, Vaz CSL, Pereira DIB, Sangioni LA (2017). Antimicrobial resistance in nontyphoidal *Salmonella* isolated from human and poultry-related samples in Brazil: 20-Year Meta-Analysis. Foodborne Pathog Dis.

[27] Costa RG, Festivo ML, Araujo MS, Reis EMF, Lázaro NS (2013). Antimicrobial susceptibility and serovars of *Salmonella* circulating in commercial poultry carcasses and poultry products in Brazil. J Food Prot.

[28] Habib I, Mohamed M-YI, Lakshmi GB, Ghazawi A, Khan M (2024). High prevalence and genomic features of multidrug-resistant *Salmonella* enterica isolated from chilled broiler chicken on retail sale in the United Arab Emirates. Int J Food Microbiol.

[29] Adetunji V, Davies A, Chisnall T, Falodun OI, Card R (2024). Epidemiology, genetic diversities and antibiotic resistance in isolates of e. coli, ESBL- e. coli and salmonella species from commercial poultry farms in ibadan, oyo state, nigeria. Preprints.

[30] WHO (2019). Critically Important Antimicrobials for Human Medicine: 6th Revision.

[31] Carattoli A (2013). Plasmids and the spread of resistance. Int J Med Microbiol.

[32] Madec J-Y, Haenni M, Nordmann P, Poirel L (2017). Extended-spectrum β-lactamase/AmpC- and carbapenemase-producing Enterobacteriaceae in animals: a threat for humans?. Clin Microbiol Infect.

[33] European Food Safety Authority, European Centre for Disease Prevention and Control (2022). The European Union One Health 2021 Zoonoses Report.

[34] Rodrigues I, Silva R, Menezes J, Machado S, Rodrigues D (2020). High prevalence of multidrug-resistant nontyphoidal *Salmonella* recovered from broiler chickens and chicken carcasses in Brazil. Braz J Poult Sci.

[35] Ministry of Health Republic of Maldives (2024). National action plan on antimicrobial resistance (AMR) 2024–2029.

